# Cost-Effectiveness of Multiple Sclerosis Disease-Modifying Therapies: A Systematic Review of the Literature

**DOI:** 10.1155/2012/784364

**Published:** 2012-12-06

**Authors:** David Yamamoto, Jonathan D. Campbell

**Affiliations:** ^1^Department of Health Systems, Management, and Policy, Colorado School of Public Health, 13001 E 17th Place, Aurora, CO 80045, USA; ^2^Department of Clinical Pharmacy, Skaggs School of Pharmacy and Pharmaceutical Sciences, University of Colorado, Anschutz Medical Campus, Aurora, CO 80045, USA

## Abstract

*Objective*. To provide a current and comprehensive understanding of the cost-effectiveness of DMTs for the treatment of MS by quantitatively evaluating the quality of recent cost-effectiveness studies and exploring how the field has progressed from past recommendations. *Methods*. We assessed the quality of studies that met our systematic literature search criteria using the Quality of Health Economic Studies validated instrument. *Results*. Of the 82 studies that met our initial search criteria, we included 22 in this review. Four studies (18%) achieved quality category 2, three studies (14%) achieved quality category 3, and 15 studies (68%) achieved the highest quality category 4. 91% of studies were simulation models. 13 studies (59%) had quality-adjusted life years (QALYs) as the primary outcome measure, included a societal perspective in the analysis, and utilized time horizons of 10 years to lifetime. *Conclusions*. To continue to improve the cost-effectiveness evidence of DMTs, we recommend: lifetime horizons, societal perspectives, and QALYs; supplemental evidence with shorter horizons, payer perspectives, and clinical outcomes to inform multiple decision makers; development of modeling and input standards for comparability; head-to-head RCTs between DMTs and long-term prospective studies; and comprehensive cost-effectiveness studies that compare all appropriate DMTs.

## 1. Introduction

Multiple sclerosis (MS) is a chronic and debilitating inflammatory autoimmune disorder of the central nervous system that afflicts approximately 400,000 people in the United States and 2.1 million people worldwide [1, 2]. MS is one of the most common contributors to neurological disability in young and middle-aged adults [3]. The peak age of onset is approximately 30 years, and the disease occurs in twice as many women as men [4]. About 85% of MS patients have the relapsing-remitting form of the disease [1]. Symptoms of MS include fatigue, impaired mobility, spasticity, pain, depression, cognitive impairment, sexual dysfunction, bowel and bladder dysfunction, vision and hearing problems, seizures, and difficulty swallowing [2]. The multifaceted symptoms of MS all are associated with economic burden, as well as an adverse impact on patients' quality of life.

Treatment of MS has advanced significantly over the past several decades. Historically, MS was treated in a solely supportive manner through symptomatic pharmacological treatment in the event of disease exacerbations (MS attacks), generally via powerful doses of short-course steroids [5]. However, starting in the 1990s the FDA approved the first disease-modifying therapies (DMTs) for the long-term treatment of the disease. These new biologics were intended to proactively manage and retard disease progression. DMTs have demonstrated favorable risk-benefit profiles for US and other jurisdictions' regulatory approval. Some DMTs have more significant benefits and risks than others. DMTs have provided a promising new means of managing this chronic and debilitating disease; however, they have also introduced greatly elevated, and rapidly increasing, costs to the treatment of MS [6]. Cost-of-illness studies in the early 2000s estimated that medication including DMTs accounted for ~7% of overall costs as reported across similar studies in Germany [7], UK [8], Sweden [9], and Canada [10]. In 2004, Kobelt found that in the US DMTs had begun to account for 34% of total costs per MS patient each year, and over half of all direct medical costs [11]. Similarly, an Italian 2004 study suggested that 77% of direct medical costs were attributed to DMTs [12]. On average DMTs cost $16,050 per MS-treated patient per year in 2004 dollars. By 2010, the current annual treatment costs had risen by two- to threefold over this 2004 average for all DMTs except mitoxantrone. The eight FDA approved DMTs as of August 2012 are characterized in Table 1.

Three review studies from 2003-4 focused on cost-effectiveness of DMTs in the treatment of MS and included multiple current therapies [3, 23, 24]. In 2004, Flachenecker published a review that comprehensively studied all DMTs available at that time [25]. In 2006, Kobelt published a review and informational article on studies of MS therapies; however, the focus was mainly methodological and did not compile and compare costs and effectiveness results in concert to inform decision makers [26]. In 2006, Reickmann published a brief review on the socioeconomic aspects of neuroimmunological diseases focusing on MS [27]. Prior reviews came to very similar conclusions suggesting use of the societal perspective, appropriate long-term modeling to reflect the chronic nature of MS, and standard endpoints and modeling to determine the cost-effectiveness of DMTs. The authors also indicated a dearth of comprehensive and uniform prospective studies, which is important in further understanding the costs and outcomes associated with different DMTs. 

Over the past ten years, there has been a lack of comprehensive and systematic reviews of multiple sclerosis cost-effectiveness studies. Previous reviews did not address DMTs approved in recent years (natalizumab or fingolimod) and none have been recent and comprehensive enough to detail the current state of the cost-effectiveness of DMTs in treating multiple sclerosis. The aim of this systematic review was to provide a current and comprehensive understanding of the cost-effectiveness of DMTs for the treatment of multiple sclerosis by quantitatively evaluating the quality of cost-effectiveness studies and exploring how the multiple sclerosis cost-effectiveness field has progressed from past recommendations. 

## 2. Methods

### 2.1. Cost-Effectiveness Analyses

Cost-effectiveness analysis (CEA) is a full form of health economic analysis, where both costs and consequences (outcomes) of health programs or treatments are examined. CEA is used in situations where decision makers are considering a limited range of options within a given field, and within a given operating budget [28]. CEA is used to compare the benefits and costs of a program, intervention, or treatment to its next best alternative in order to determine whether it is of sufficient value to adopt or reimburse [29]. The primary output of a cost-effectiveness study is the incremental cost-effectiveness ratio (ICER), which compares two alternative interventions' average costs (*C*_1_ and *C*_2_) and effects (*E*_1_ and *E*_2_) in the form of the following ratio:

(1) $$\begin{array}{c} \frac{\left(C_{1}-C_{2}\right)}{\left(E_{1}-E_{2}\right)}. \end{array}$$

The effectiveness measure chosen should properly reflect a final output, rather than a secondary or intermediate output. However, the most germane consideration is whether the measure is relevant given the objectives of the decision maker concerned [28]. When there are multiple objectives of treatments or interventions, an array of differential achievements along each dimension may be presented for the alternative interventions. These data can then be used at the discretion of the decision maker to most appropriately address their unique situation [28]. This form of evaluation is referred to as cost-consequence analysis (CCA). Another form of CEA, cost-utility analysis (CUA), seeks to incorporate the weighted measures of all relevant outcomes in one measure based upon overall utility. The most common outcome measure used is the quality-adjusted life year (QALY), which estimates the alternative-specific survival and assigns utility weights ranging from zero (death) to one (perfect health) for each life year [30]. 

CEA studies follow a decision-analytic modeling (DAM) approach where currently available evidence concerning the effectiveness and costs of alternative healthcare interventions is synthesized in order to inform decision makers about the relative value of competing alternatives [31]. DAM helps decision makers to formulate as informed decisions as possible under conditions of cost and effectiveness uncertainty and resource scarcity. There are two main subbranches of DAM of interest for CEA of DMTs in MS: simulation models and patient-level trials. Simulation models can either be based upon patient cohorts or individual patient-level data. The most common type of cohort simulation model (CSM) in MS is the Markov model, where cohorts transition among multiple disease states via assigned probabilities calculated for specific time periods (cycles). Each disease state is assigned costs and outcomes in order to calculate outputs [28]. Patient-level trials are typically conducted within one randomized trial or within one observational study where both clinical and economic analyses are performed using the patient-level data.

### 2.2. Literature Search

**Figure 1:**
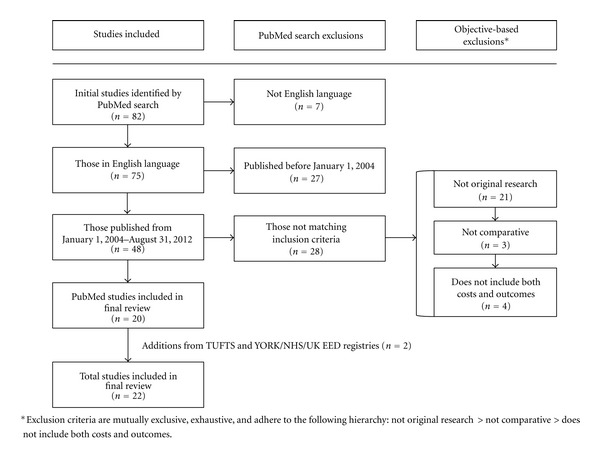
Flowchart of systematic search methodology and yield.

We performed a systematic search in September of 2012 using MEDLINE (PubMed) querying for the MeSH term, “cost-benefit analysis,” and the general search term, “multiple sclerosis.” The MeSH term “cost-benefit analysis” includes the following nested entry terms: cost-benefit, cost-effectiveness, and benefits and costs. There is no other MeSH term comprehensive enough to require elements of both costs and health outcomes. To control for the impact of including DMT generic and brand names in the query, we included (mitoxantrone OR interferon OR glatiramer acetate OR natalizumab OR fingolimod OR avonex OR betaseron OR extavia OR rebif OR novantrone OR copaxone OR gilenya OR tysabri) in addition to the initial search terms as part of an additional search for comparative purposes. We utilized PubMed search filters to limit the initial search to articles published in the English language from January 1, 2004 to August 31, 2012. In addition to the PubMed filters applied, we imposed a three-point exclusion criterion to the search results. The exclusion criteria were mutually exclusive, exhaustive, and hierarchical in the following manner (see Figure 1):

1. not original research;
2. not comparative;
3. does not include both costs and outcomes.

If an article was excluded under the first criterion, it would not be further scrutinized for adherence to the second or third criteria, and if it adhered to the first but not to the second, it would not be further scrutinized for adherence to the third criterion. Those that reached this final exclusion criterion and adhered to all three criteria were included in the final systematic review database. To ensure the comprehensiveness of our database, we performed two additional searches using the same queries and criteria via the Tufts Medical Center Cost-Effectiveness Analysis Registry (TUFTS) and the National Health Service Economic Evaluation Database (NHSEED). Additional articles that were not found in the initial search and adhered to our three-point criterion were added to the final database.

### 2.3. Quality Assessment of Health Economic Analyses

We quantitatively evaluated the quality of the MS DMT cost-effectiveness studies in a systematic and transparent manner through the use of the 16-item Quality of Health Economic Studies (QHES) validated instrument [32]. Both authors independently applied the QHES scoring instrument to each of the articles included in the final database and came to an agreement on all scores for each study. The final reconciled scores were reported. Total QHES scores range from 0 to 100, with 100 representing a perfect score. Four categories have been established to stratify the studies from lowest to highest quality: lowest quality category 1 (total score: 0–25), quality category 2 (total score: 25.1–50), quality category 3 (total score: 50.1–75), and highest quality category 4 (75.1–100). 

### 2.4. QHES Subdomains

We identified four main topics to focus our discussion of the composition and quality of the included cost-effectiveness studies: (i) model structure (QHES items 1, 2, 8, 12, and 13), (ii) model inputs (QHES items 3, 7, 9, 10, and 11), (iii) results/conclusions (QHES items 6, 11, and 15), and (iv) study integrity (QHES items 5, 13, 14, and 16) [32]. Item 11 is included in both the model inputs and results/conclusions subdomains. Appropriate choice of model structure is of paramount importance in determining the reach and applicability of the results and conclusions of CEA studies. In addition, transparency and justification of the model framework and its assumptions are crucial to enable an unbiased peer review process as well as in determining the usability and repeatability of the study and its findings [30]. Appropriate acknowledgment of the type of data abstraction employed and justification for the methodology allows users to replicate and validate the analysis [30]. It is important that the model inputs are appropriately matched to the model structure employed, specifically with respect to the audience it is meant to inform (study perspective) and the analytical time horizon employed, in order to properly advance the knowledge pool that decision makers draw from in developing protocols for the treatment of MS. The main results of CEA studies are presented via the ICER. This ratio describes the incremental cost of an intervention for each additional unit of benefit. This form of presenting CEA study results allows for a standardized interpretation of cost-effectiveness between comparators and is the cornerstone of quality studies in this field. A negative ICER indicates that one of the alternatives is more effective and less costly than the other and is considered to dominate the other. A positive ICER indicates that one alternative is both less costly and less effective or more costly and more effective. An intervention is considered cost-effective if it dominates its comparator or if it is both more (or less) costly and effective, while falling below the decision maker's willingness-to-pay threshold [30]. The resulting ICER and conclusion of a CEA study are only useful if it is justified based upon the study results. The integrity of a study relies on an explicit transparency with respect to uncertainty, limitations, assumptions, and bias. Clearly describing uncertainty with respect to individual variables in isolation (one-way sensitivity analysis), as well as in conjunction (multiway sensitivity analysis, probabilistic sensitivity analysis, etc.), informs decision makers as to which inputs have the greatest impact on results and indicates areas for future research. 

## 3. Results

### 3.1. Literature Search Results

Of the 82 total studies that met our initial MEDLINE search criteria [1, 3, 4, 23–25, 31, 33–107], we included 20 in our review (Figure 1) [1, 33, 35, 38, 39, 48, 52, 55, 58, 59, 62, 66, 77, 78, 80, 84, 100, 101, 106, 107]. With respect to the PubMed filter exclusions, seven of 80 studies were not published in English and 27 others were published before January 1, 2004. The most common reason for excluding studies matching our initial search queries and filters was not reporting original research (i.e. reviews, methods papers, letters, and editorials were excluded). 21 studies were excluded due to this primary objective-based exclusion [3, 23, 25, 31, 34, 43–45, 49, 53, 60, 67, 69, 72, 75, 86, 87, 91, 97, 99, 105]. Three other studies were deemed not comparative [4, 54, 83] and an additional four studies did not include both costs and outcomes [36, 65, 71, 88]. We located two articles [108, 109] in the TUFTS and NHSEED databases that met all of our criteria, resulting in a final total of 22 articles included in our review.

### 3.2. Results by Model Type

Table 2 contains characteristics of each study, including details pertaining to the model type chosen. 20 of the 22 studies were simulation models: 19 of these 20 were cohort simulation models (CSM) [1, 33, 35, 39, 48, 52, 58, 59, 62, 66, 77, 78, 80, 84, 100, 101, 106–108] and one was a patient-level simulation [55]. Of the 19 CSM, 14 were Markov models [35, 48, 52, 58, 59, 62, 77, 78, 84, 100, 101, 106–108]. Within the Markov models employed, four studies utilized one-month transition periods [35, 48, 59, 84], two studies utilized three-month transition periods [62, 108], two used six-month transition periods [106, 107], and six studies utilized one-year transition periods [52, 58, 77, 78, 100, 101].

We identified two patient-level trial-based studies: one based on an open-labeled head-to-head clinical trial [109] and one using data from a retrospective multivariate cohort analysis [38].

### 3.3. Results by Geographic Region and Perspective

12 of the 22 studies were based upon data from the USA [1, 33, 35, 38, 39, 48, 55, 77, 80, 84, 100, 106]. Of these USA studies, seven were performed under a payer/health care system perspective [1, 33, 38, 39, 55, 80, 100], four were from a societal perspective [35, 77, 84, 106], and one included both perspectives [48]. There were two studies based upon data from the UK, one of which was from a societal perspective [52] and one was from a payer perspective [101]. There were two studies based upon data from Italy, one of which was from the payer perspective [109] and one included both societal and payer perspectives [66]. Two studies were derived from Swedish data and were performed from a societal perspectives [62, 107]. One study was derived from Canadian data and was performed from both societal and payer perspectives [58]. One study was derived from French data and was performed from both societal and payer perspectives [108]. One study was derived from Balkan data and was performed from a societal perspective [59]. One study was derived from German data and was performed from a societal perspective [78]. A total of nine studies utilized a payer perspective in their base-case analysis [1, 33, 38, 39, 55, 80, 100, 101, 109], nine studies utilized a societal perspective in their base-case analysis [35, 52, 59, 62, 77, 78, 84, 106, 107], and four studies included both perspectives in their base-case analysis [48, 58, 66, 108].

### 3.4. Results by DMT Type

Of the included studies, 16 included an interferon product [1, 33, 35, 38, 39, 52, 55, 58, 59, 66, 77, 78, 84, 100, 106, 107], 12 included glatiramer acetate [1, 33, 35, 38, 39, 48, 52, 59, 77, 78, 84, 100], six included natalizumab [33, 39, 48, 52, 62, 80], two included mitoxantrone [101, 109], and one included fingolimod [80]. The cost-effectiveness of each DMT varied greatly depending upon the study design, perspective, and treatment comparisons chosen. However, natalizumab was found to be cost-effective or dominant in all studies it appeared in [33, 39, 48, 52, 62, 80], with one exception being the lifetime cost-effectiveness study from a healthcare perspective by Earnshaw et al. [48]. Mitoxantrone was not compared to another DMT in either of the studies in which it was included [101, 109]. 

### 3.5. Results by Primary Outcome and Time Horizon

The most prominent primary effectiveness outcome chosen was QALYs, which was represented in 13 of the 22 studies [35, 48, 52, 59, 62, 66, 77, 84, 100, 101, 106–108]. The next most prevalent primary effectiveness outcome was relapses avoided, which was represented in six studies [1, 33, 39, 55, 78, 80]. Two studies included relapse rate reduction as a primary outcome [38, 109]. Reduction in EDSS score [109], cost to health plan [39], relapse free days gained [55], and quality-adjusted monosymptomatic life years (QAMLY) [58] were chosen as primary outcomes each in one study. The studies with QALYs as the primary outcome had time horizons that ranged from 10 years to lifetime. The studies with relapses avoided as the primary outcome had time horizons of either two or four years.

### 3.6. Quality Assessment of Health Economic Analyses Results

Table 1 presents the QHES scores for each cost-effectiveness study. No studies were assigned quality category 1, four studies were assigned quality category 2 [33, 38, 59, 109], three studies were assigned quality category 3 [58, 66, 108], and 15 studies were assigned the highest quality category 4 [1, 35, 39, 48, 52, 55, 62, 77, 78, 80, 84, 100, 101, 106, 107].

### 3.7. QHES Subdomain Results

#### 3.7.1. Model Structure

In 21 of the 22 studies, the objective was “presented in a clear, specific, and measurable manner” (QHES item 1) [1, 35, 38, 39, 48, 52, 55, 58, 59, 62, 66, 77, 78, 80, 84, 100, 101, 106–109]. 12 studies appropriately stated and justified the perspective employed (QHES item 2) [1, 39, 52, 55, 78, 80, 84, 100, 101, 106–108]. 16 studies justified the time horizon chosen as appropriate to capture all important and relevant outcomes (QHES item 8) [1, 35, 39, 48, 52, 55, 59, 62, 66, 78, 80, 84, 100, 101, 106, 107]. In 14 studies the economic model, methods, and analysis were transparent and repeatable (QHES item 12) [1, 35, 48, 52, 58, 62, 77, 78, 84, 100, 101, 106–108]. 14 studies properly justified the model chosen (QHES item 13) [1, 35, 39, 48, 52, 55, 62, 77, 80, 100, 101, 106–108].

#### 3.7.2. Model Inputs: Cost and Effectiveness Measures

Inputs were drawn from the best available source in 20 of the 22 studies (QHES item 3) [1, 33, 35, 38, 39, 48, 52, 55, 58, 62, 66, 77, 78, 80, 84, 100, 101, 106–108]. The data abstraction methodology was adequately stated and repeatable in 20 studies (QHES item 7) [1, 35, 39, 48, 52, 55, 58, 59, 62, 66, 77, 78, 80, 84, 100, 101, 106–109]. The estimation of costs was appropriate and repeatable in 20 studies (QHES item 9) [1, 33, 35, 39, 48, 52, 55, 58, 59, 62, 66, 77, 78, 80, 84, 100, 101, 106–108]. In 16 studies the primary outcome measure was clearly stated and negative outcomes were included, or justification was given for their omission from the analysis (QHES item 10) [1, 35, 39, 48, 52, 55, 58, 66, 78, 80, 84, 100, 101, 106, 107, 109]. 19 studies chose valid primary outcome measures and justified them adequately (QHES item 11) [1, 33, 35, 38, 39, 48, 52, 55, 62, 66, 77, 78, 80, 84, 100, 101, 106, 107, 109].

#### 3.7.3. Results and Conclusions

20 of the 22 studies performed an incremental analysis for costs and outcomes between alternatives (QHES item 6) [1, 33, 35, 39, 48, 52, 55, 58, 59, 62, 66, 77, 78, 80, 84, 100, 101, 106–108]. 15 studies provided stated conclusions that were justified and based upon study results (QHES item 15) [33, 35, 38, 52, 58, 62, 66, 77, 80, 84, 100, 101, 106–108].

#### 3.7.4. Study Integrity: Uncertainty, Limitations, Assumptions, Bias, and Funding

20 of the 22 studies addressed uncertainty (QHES item 5) [1, 33, 35, 38, 39, 48, 52, 55, 58, 59, 62, 66, 77, 78, 80, 84, 100, 101, 107, 109]. 14 studies explicitly stated and justified the assumptions and limitations of the chosen model (QHES item 13) [1, 35, 39, 48, 52, 55, 62, 77, 80, 100, 101, 106–108]. Nine studies explicitly discussed the magnitude and direction of potential biases (QHES item 14) [48, 52, 62, 77, 100, 101, 106–108]. 21 studies provided a statement disclosing the source of funding for the study (QHES item 16) [1, 33, 35, 38, 39, 48, 52, 55, 58, 59, 62, 66, 77, 78, 80, 84, 100, 101, 106–108].

## 4. Discussion

Clinical guidelines for the treatment of MS with DMTs remain underdeveloped and lacking in comprehensive understanding and consensus regarding what DMT should be used for what type of MS patient. The American Academy of Neurology (AAN) and the MS Council for Clinical Practice Guidelines have not published comprehensive guidelines including all current DMTs for the US since 2002 (guidelines were reaffirmed in 2008) [110]. In the UK, the National Institute for Clinical Excellence (NICE) relies on guidelines for the treatment of MS that were published in 2003 [111]. In parallel to the development of current and accurate clinical guidelines is the need for quality and consistency in CEA of DMTs for MS. We compare our systematic review findings with past recommendations and highlight areas of progress and those in need of further development.

There was a preponderance of simulation models within our included studies. Simulation modeling allows for the projection of short-term data to reflect the chronic nature of MS. The use of simulation models in lieu of long-term DMT studies indicates progress in the field and is consistent with previous recommendations regarding the use of simulation modeling when long-term cost and outcome studies are lacking. The large majority of these simulation models employed a Markov structure, which allows for long-term analysis, up to lifetime in scope. Markov modeling also allows for transition between disease states for cohorts of patients, which reflects the natural disease progression within MS. This further indicates a positive trajectory in the cost-effectiveness evaluation of DMTs for MS.

The past recommendations supporting use of the long-term time horizons and societal perspectives harmonize well with the use of QALYs as the primary outcome measure, as was suggested by Kobelt [26]. The majority of studies utilized long-term time horizons. More than half of the studies included a societal perspective in their base-case analysis and utilized QALYs as their primary outcome measure indicating progress, albeit with room for improvement in adhering to past recommendations. However, there were a significant number of studies that utilized a two- or four-year time horizon, payer perspective, and relapses avoided as their primary outcome measures. The short-term time horizon studies provide evidence for example to USA insurers who may have 2- to 4-year average insured time horizons for their populations. Past recommendations can be advanced by suggesting that CEA studies on DMTs for MS should primarily adopt a long-term time horizon, societal perspective, and QALYs as the fundamental recommendation, with the option to supplement the study with an added analysis using short-term time horizons, payers perspectives, and clinical effectiveness (or safety) measures such as relapses avoided. A cost-consequences approach would allow for more than one effectiveness (or safety) measure to be compared across DMTs and should be considered depending upon the decision makers' needs.

The assumptions employed in the simulation models were diverse and inconsistent between studies indicating much room for improvement in conforming to past recommendations with respect to consistency in modeling. This diversity is a factor in the wide range of cost-effectiveness estimates for the same DMTs across studies. The generally poor performance of studies in explicitly stating and properly justifying model assumptions, discussing the magnitude and direction of potential biases, employing transparent and repeatable models/methods, and justifying their choice of model (QHES items 12, 13, and 14) further indicates a lack of adherence to proper guidelines within the field for consistent and appropriate modeling methodology. We call for more efforts in the standardization of cost-effectiveness studies within MS and for studies to provide rationale for why the design and assumptions may differ from previous cost-effectiveness studies.

The lack of head-to-head randomized controlled trials (RCTs) between DMTs and absence of long-term observational data serve to drive the need for cost-effectiveness studies to employ multiple assumptions since the appropriate long-term comparative data do not exist. The currently available RCT data are generally specific to individual DMTs and are based upon different patient populations and different study characteristics such as treatment adherence, dropout rates, and adverse outcomes. Therefore, the results of individual studies are contingent upon which RCT data are employed, along with the assumptions included in the model. This heterogeneity makes comparing the results across studies difficult. More comparative head-to-head RCTs among DMTs and prospective observational studies are needed and will generate less heterogeneity in cost-effectiveness model structures and inputs yielding less uncertainty in cost-effectiveness results. It is important that proper guidelines for cost-effectiveness modeling [112] are utilized by researchers to maintain consistency and comparability within the field. Progress of this order is necessary to satisfy the recommendation that standard modeling is employed across studies, which we found to still be largely unfulfilled in recent studies.

Finally, there was a lack of cost-effectiveness studies on fingolimod. There is a need for studies including fingolimod as a comparator, specifically coupled with traditional DMTs (interferons and glatiramer acetate) to determine this new treatment's value as a therapy option in MS. Given the high annual treatment cost of fingolimod, the cost-effectiveness evidence as compared to other DMTs becomes even more important. There were no studies that included all approved DMTs as comparators indicating a need for a comprehensive study including all appropriate DMTs for particular MS patient populations. A comprehensive cost-effectiveness study of this nature would help to alleviate the problems of comparing cost-effectiveness across different studies that employ different methodologies and study assumptions.

## 5. Conclusion

The cost-effectiveness body of evidence of DMTs for the treatment of MS has shown progress in responding to the recommendation of past reviews, while there remains room for improvement in many areas. The area of greatest advancement is in the use of simulation models that represent the chronic nature of the disease. This appears to be the dominant trend in current studies. To a lesser degree, the field has shown progress in adhering to the recommendation that long-term time horizons, societal perspectives, and QALYs are utilized, albeit with room for further improvement. We recommend that studies utilize lifetime horizons, societal perspectives, and QALYs as the primary standards in CEA studies of DMTs for MS, with the option to supplement the base-case analysis by including short-term horizons, payer perspectives, and a cost-consequences approach. This recommendation conforms to the suggestions of past reviewers, while adding the ability for individual studies to inform multiple decision makers. There is a great need for improvement in the standardization of modeling procedures and data inputs. We recommend that modeling and input assumption standards are developed within MS cost-effectiveness studies to aid comparability across future studies. The recommendation to perform head-to-head RCTs between DMTs and collect long-term prospective observational data would improve study consistency in the future. Finally, we recommend that comprehensive studies comparing all approved DMTs in concert are performed to help control for the inconsistencies between studies and provide meaningful results for decision makers.

## Figures and Tables

**Table 1:** Characteristics of disease-modifying therapies for multiple sclerosis.

DMT brand name (Generic name)	Manufacturer	FDA approval year	Dose frequency administration [5]	2010 Annual Tx cost [13, 14]	Significant risks listed in package insert
Betaseron [15] (IFN beta-1b)	Bayer Health Care Pharmaceuticals, Inc	1993	250 ug 2 days SC	$38,369	
Avonex [16] (IFN beta-1a)	Biogen Idec, Inc	1996	30 ug weekly IM	$38,532	
Copaxone [17] (Glatiramer acetate)	Teva Neuroscience, Inc	1996	20 mg daily SC	$42,940	
Novantrone [18] (Mitoxantrone)	Novartis Pharmaceuticals Corporation	2000	12 mg/m^2^ 3 months IV	$6,344	Cardiotoxicity
Rebif [19] (IFN beta-1a)	EMD Serono, Inc	2000	44 ug 3x weekly SC	$38,646	
Tysabri [20] (Natalizumab)	Elan Pharmaceuticals, Inc and Biogen Idec, Inc	2004	300 mg 4 weeks IV	$40,426	Increased risk of PML, Elevated risk for infections
Extavia [21] (IFN beta-1b)	Novartis Pharmaceuticals Corporation	2009	250 ug 2 days SC	$38,368	
Gilenya [22] (Fingolimod)	Novartis Pharmaceutical Corporation	2010	0.5 mg daily oral	$47,944	Cardiotoxicity, Elevated risk for infections

IFN: Interferon; IM: intramuscular; IV: intravenous; PML: progressive multifocal leukoencephalopathy; SC: subcutaneous; Tx: treatment.

**Table 2:** Cost-effectiveness analyses of multiple sclerosis disease modifying therapies (2004–2012).

First author year	Model type country perspective	Study pop time horizon	Comparators	Primary effectiveness outcome	Results	Stated conclusion	QHES score	Sponsor
Prosser 2004 [84]	CSM^1^ US societal	Non-primary progressive MS 10 years	(1) IFNB-1a (IM) (2) IFNB-1b (3) GA (4) No Tx	QALYs	1 versus 4: Not CE (>$1,800,000/QALY) 2 & 3 dominated versus 4	1–3 less CE than 4 under a wide range of assumptions.	87	National MS Society

Iskedjian 2005 [58]	CSM^1^ Canadian MoH/societal	SDE at risk for CDMS 15 yrs	(1) IFNB-1a (IM) + MPS (iv) (2) MPS (iv)	QAMLY	$227,586/QAMLY (MoH) $189,286/QAMLY (SoC)	1 is a reasonably CE approach to Tx of patients with a SDE. ICER improves if Tx is initiated in pre-CDMS.	69	Biogen Idec, Inc.

Perini 2006 [109]	OLH2HCS Italian payer (NS)	SPMS 2 years	(1) Mitoxantrone (2) CP	Relapse rate decrease/EDSS decrease	1: 88%/0.9 (€8171) 2: 86.4%/0.9 (€5097)	2 should be considered as a first-line rescue therapy for MS	34	Not stated

Bell 2007 [35]	CSM^1^ US societal	RRMS Lifetime	(1) GA (2) IFNB-1a (IM) (3) IFNB-1a (SC) (4) IFNB-1b (5) Supportive	QALYs	1 versus 5: $258,465/QALY 2 versus 5: $337,968/QALY 3 versus 5: 416,301/QALY 4 versus 5: $310,691/QALY	1 is the best DMT and resulted in better outcomes than 5 alone.	90	Teva Neuroscience, Inc.

Gani 2008 [52]	CSM^1^ UK societal	HARRMS 30 years	(1) Natalizumab (2) IFNB (pooled) (3) GA (4) Supportive	QALYs	1 versus 2: *£*2300/QALY 1 versus 3: *£*2000/QALY 1 versus 4: *£*8200/QALY	1 is more CE than 2, 3 and 4 for HARRMS.	100	Biogen Idec, Inc.

Kobelt 2008 [62]	CSM^1^ Swedish societal	RRMS 20 years	(1) Natalizumab (2) DMDs (composite)	QALYs	1 dominates 2	For this population, 1 provides an additional health benefit at a similar cost to 2.	90	Biogen Idec Inc./Elan Pharma.

Castelli-Haley 2009 [38]	RMCA US payer (NS)	ITT: CDMS, GA or IFNB-1b Rx, Ins. cov. CU: ITT + no other DMT and Rx in last 28 days of period 2 years	(1) GA (2) IFNB-1b	Risk of Relapse Avg. total medical costs	ITT: 5.31% (1), 13.54% (2) CU: 2.09% (1), 10.91% (2) $48,130 (1), $53,157 (2)	RRMS patients treated with 1 have significantly lower relapse rate. Costs are lower for 1 in CU cohort.	43	Teva Neuroscience, Inc.

Chiao 2009 [39]	CSM^2^ US payer	DMT candidates with relapsing MS 2-years (static)	(1) Natalizumab (2) IFNB-1a (IM) (3 IFNB-1b (4) GA (5) INFB-1a (SC)	Relapses avoided	1 versus 2: $23,029/RA 1 versus 3: $24,452/RA 1 versus 4: $20,671/RA 1 versus 5: $20,403/RA	1 was the most CE.	78	Biogen Idec, Inc./Elan Pharma.

Earnshaw 2009 [48]	CSM^1^ US healthcare (also societal)	RRMS Lifetime	(1) Natalizumab (2) GA (3) Supportive	QALYs	Healthcare: 1 versus 3: $606,228/QALY 2 versus 3: $496,222/QALY Societal: 1 & 2 cost saving versus 3	1 and 2 are associated with increased benefits compared with 3 at higher costs.	88	Teva Neuroscience, Inc.

Goldberg 2009 [1]	CSM^3^ US payer	RRMS 2 years	(1) GA (2) IFNB-1a (IM) (3) IFNB-1a (SC) (4) IFNB-1b (5) No Tx	Relapses avoided	1 versus 5: $88,310/RA 2 versus 5: $141,721/RA 3 versus 5: $80,589/RA 4 versus 5: $87,061/RA	1, 3 and 4 represent the most CE DMDs for Tx of RRMS.	86	EMD Serono, Inc

Guo 2009 [55]	PLS^4^ US payer	Relapsing MS 4 years	(1) IFNB-1a (SC) (2) IFNB-1a (IM)	Relapses avoided/ Relapse free days gained	1 versus 2: $10,755/RA $232/relapse free day	1 versus 2 yields greater health benefits over 4 years at a reasonable cost.	78	EMD Serono, Inc

Jankovic 2009 [59]	CSM^1^ Balkan societal	RRMS 40 years	(1) GA (SC) (2) IFNB-1a (IM) (3) IFNB-1a (SC) (4) IFNB-1b (5) Supportive	QALYs	>$20,000,000/QALY (1–4 versus 5)	IMT of RRMS in a Balkan country is not CE.	46	Serbian Ministry of Science and Ecology

Kobelt 2009 [108]	CSM^1^ French societal (also payer)	Relapsing forms 20 years	(1) DMTs (2) No DMT	QALYs	1 versus 2: €15,385/QALY (Fully treated with DMT)	Cost increase with DMTs is moderate for health gained.	71	Authors declare none

Lazzaro 2009 [66]	CSM^5^ INHS/Italian societal	CIS patients 25 years	IFNB-1b: (1) At CIS (2) At CDMS	QALYs	INHS: €2,575 1 versus 2 Societal: 1 dominates 2	1 significantly delays conversion to CDMS and is economically advantageous.	75	Bayer Schering Pharma, Italy

Tappenden 2009 [100]	CSM^1^ US payer (also CMS)	Medicare beneficiaries with MS 50 year	(1) IFNB-1a (PA) (2) IFNB-1a (SA) (3) IFNB-1a (22 ug) (4) IFNB-1a (44 ug) (5) IFNB-1b (6) GA (7) Supportive	QALYs	1 versus 7: $66–234k/QALY 2 versus 7: $60–218k/QALY 3 versus 7: $120–199k/QALY 4 versus 7: $79–172k/QALY 5 versus 7: $91–169k/QALY 6 versus 7: $122–312k/QALY	Suggests prudent use of a discontinuation rule may improve CE.	100	USDHHS AHRQ

Bakshai 2010 [33]	CSM^2^ US managed care payer	Relapsing forms receiving IMT 2 years	(1) Natalizumab (2) IFNB-1a (IM) (3) IFNB-1b (4) GA (5) IFNB-1a (SC)	Relapses avoided	1 versus 2: $23,029/RA 1 versus 3: $24,452/RA 1 versus 4: $20,671/RA 1 versus 5: $20,403/RA	1 is relatively CE and adds a new option for those patients for whom conventional Tx was unsuccessful.	50	Not funded

Nuijten 2010 [78]	CSM^1^ German societal	RRMS 4 years	(1) IFNB-1a (SC) (2) IFNB-1a (IM) (3) IFNB-1b (4) GA (5) No Tx	Relapses avoided	1 versus 5: €51,250/RA 2 versus 5: €133,770/RA 3 versus 5: €54,475/RA 4 versus 5: €71,416/RA	1 versus 5 had favorable overall CE compared with all other available DMDs for the Tx of RRMS.	79	Merck Pharma

Tappenden 2010 [101]	CSM^1^ UKNHS/PSS	SPMS Lifetime	(1) AHSCT (2) Mitoxantrone	QALYs	1 versus 2: *£*2783 to Dominated (Cost/QALY)	1 could potentially achieve an acceptable CE, however RCTs are needed to confirm this.	100	No commercial or research funding

Noyes 2011 [77]	CSM^1^ US societal (NS)	RRMS and SPMS 10 years	(1) IFNB-1a (IM) (2) IFNB-1b (3) GA (4) IFNB-1a (SC) (5) Supportive	QALYS	1–4 versus 5: All far exceeded $800,000/QALY	DMTs resulted in small health gains for the cost. Starting DMTs earlier resulted in more favorable CE results.	83	Biogen, Boston Scientific, NIH, NMSS, and USDOD

O'Day 2011 [80]	CSM^3^ US managed care payer	Relapsing MS 2 years	(1) Natalizumab (2) Fingolimod	Relapses avoided	1 dominates 2	1 dominates 2 in terms of incremental cost per relapse avoided.	86	Biogen Idec, Inc.

Caloyeras 2012 [107]	CSM^1^ Swedish Societal	First initial event suggestive of MS and CDMS 50 years	(1) IFNB-1b early treatment (2) IFNB-1b delayed treatment	QALYS	1 dominates 2	Early treatment improved patient outcomes while controlling costs. 1 dominates 2	99	Abt Bio Pharma Solutions, Inc.

Pan 2012 [106]	CSM^1^ US Societal	RRMS 70 years	(1) IFNB-1b early treatment (2) Placebo, market basket after 5 years	Life-Years Gained; QALYS	1 versus 2: $30,967/LY; $46,357/QALY	Treatment during early disease phase increased patient life-years and QALYs. Early treatment with IFNB-1b likely cost-effective	90	Bayer Health-Care Pharmaceuticals

Models: ^1^Markov, ^2^Cost-effectiveness/budget impact, ^3^Average patient simulation, ^4^Discrete event simulation, ^5^Open cohorts epidemiology model.

Comparators: No Tx: No physician care, Supportive: Symptom management alone.

Acronyms (alphabetical): AHSCT: Autologous haematopoietic stem cell transplantation; CE: Cost effective; CDMS: Clinically diagnosed multiple sclerosis; CIS: Clinically isolated syndrome; CP: Cyclophosphamide; CSM: Cohort simulation model; CU: Continuous use; DMD: Disease-modifying drug; DMT: Disease-modifying therapy; HARRMS: Highly active relapsing-remitting multiple sclerosis; IM: Intramuscular; IMT: Immunomodulatory treatment; INHS: Italian National Health Service; ITT: Intent to treat; MLY: Monosymptomatic life years gained; MoH: Ministry of Health; MPS: Methylprednisolone; NS: Not stated; OLH2HCS: Open-labeled head-to-head clinical study; PA: Physician-administered subcutaneous; PLS: Patient-level simulation; PMPM: Per member per month; PSS: Personal Social Services; QAMLY: Quality-adjusted monosymptomatic life years gained; RA: relapse avoided; RMCA: Retrospective multivariate cohort analysis; RRMS: Relapsing-remitting multiple sclerosis; SA: Self-administered subcutaneous; SC: Subcutaneous; SDE: Single demyelinating event; SoC: Societal; SPMS: Secondary progressive multiple sclerosis; UKNHS: United Kingdom National Health Service.

## References

[1] Goldberg LD, Edwards NC, Fincher C, Doan QV, Al-Sabbagh A, Meletiche DM (2009). Comparing the cost-effectiveness of disease-modifying drugs for the first-line treatment of relapsing-remitting multiple sclerosis. *Journal of Managed Care Pharmacy*.

[2] Zwibel HL, Smrtka J (2011). Improving quality of life in multiple sclerosis: an unmet need. *The American Journal of Managed Care*.

[3] Phillips CJ (2004). The cost of multiple sclerosis and the cost effectiveness of disease-modifying agents in its treatment. *CNS Drugs*.

[4] Olofsson S, Wickstrom A, Hager Glenngard A, Persson U, Svenningsson A (2011). Effect of treatment with natalizumab on ability to work in people with multiple sclerosis: productivity gain based on direct measurement of work capacity before and after 1 year of treatment. *BioDrugs*.

[5] Multiple Sclerosis Association of America http://www.msassociation.org/about_multiple_sclerosis/treating/.

[6] Schafer JA, Gunderson BW, Gleason PP (2010). Price increases and new drugs drive increased expenditures for multiple sclerosis. *Journal of Managed Care Pharmacy*.

[7] Kobelt G, Lindgren P, Smala A (2001). Costs and quality of life in multiple sclerosis. An observational study in Germany. *HEPAC Health Economics in Prevention and Care*.

[8] Kobelt G, Lindgren P, Parkin D (2000). Costs and quality of life in multiple sclerosis: a cross-sectional observational study in the United Kingdom. *EFI Research Reports*.

[9] Henriksson F, Fredrikson S, Masterman T, Jönsson B (2001). Costs, quality of life and disease severity in multiple sclerosis: a cross-sectional study in Sweden. *European Journal of Neurology*.

[10] Grima DT, Torrance GW, Francis G, Rice G, Rosner AJ, Lafortune L (2000). Cost and health related quality of life consequences of multiple sclerosis. *Multiple Sclerosis*.

[11] Kobelt G, Berg J, Atherly D, Hadjimichael O (2006). Costs and quality of life in multiple sclerosis: a cross-sectional study in the United States. *Neurology*.

[12] Russo P, Capone A, Paolillo A (2004). Cost-analysis of relapsing-remitting multiple sclerosis in Italy after the introduction of new disease-modifying agents. *Clinical Drug Investigation*.

[13] Staff Thomson PDR (2010). *Red Book: Pharmacy's Fundamental Reference*.

[15] Pharmaceuticals BH Betaseron Prescribing Information. http://berlex.bayerhealthcare.com/html/products/pi/Betaseron_PI.pdf.

[16] Biogen I Avonex Prescribing Information. http://www.avonex.com/pdfs/guides/Avonex_Prescribing_Information.pdf.

[17] Teva Neuroscience I Copaxone Prescribing Information. http://www.sharedsolutions.com/redirect/PrescribingInformation.pdf.

[18] National Center for Biotechnology Information Novantrone Black Box Warning. http://www.ncbi.nlm.nih.gov/books/NBK50576/.

[19] Serono I EMD Rebif Prescribing Information. http://www.emdserono.com/cmg.emdserono_us/en/images/rebif_tcm115_19765.pdf.

[20] Elan Pharmaceuticals I Tysabri Prescriber Information. http://www.tysabri.com/pdfs/I61061-13_PI.pdf>.

[21] Corporation NP Extavia Important Product Information (Prescribing Information). http://www.pharma.us.novartis.com/product/pi/pdf/extavia.pdf.

[22] Coporation NP Gilenya Prescribing Information. http://www.pharma.us.novartis.com/product/pi/pdf/gilenya.pdf.

[23] Amato MP (2004). Pharmacoeconomic considerations of multiple sclerosis therapy with the new disease-modifying agents. *Expert Opinion on Pharmacotherapy*.

[24] Flachenecker P, Rieckmann P (2003). Early intervention in multiple sclerosis: better outcomes for patients and society?. *Drugs*.

[25] Flachenecker P, Rieckmann P (2004). Health outcomes in multiple sclerosis. *Current Opinion in Neurology*.

[26] Kobelt G (2006). Health economic issues in MS. *International MS Journal / MS Forum*.

[27] Rieckmann P (2006). Socio-economic aspects of neuroimmunological diseases. *Journal of Neurology*.

[28] Drummond Michael SMJ F, Torrance George W, O.'Brien Bernie J, Stoddart Greg L (2005). *Methods for the Economic Evaluation of Health Care Programmes*.

[29] Sloan F (1996). *Valuing Health Care: Costs, Benefits, and Effectiveness of Pharmaceuticals and Other Medical Technologies*.

[30] Campbell JD, Spackman DE, Sullivan SD (2008). Health economics of asthma: assessing the value of asthma interventions. *Allergy*.

[31] Philips Z, Bojke L, Sculpher M, Claxton K, Golder S (2006). Good practice guidelines for decision-analytic modelling in health technology assessment: a review and consolidation of quality assessment. *PharmacoEconomics*.

[32] Chiou CF, Hay JW, Wallace JF (2003). Development and validation of a grading system for the quality of cost-effectiveness studies. *Medical Care*.

[33] Bakhshai J, Bleu-Lainé R, Jung M (2010). The cost effectiveness and budget impact of natalizumab for formulary inclusion. *Journal of Medical Economics*.

[34] Becker RV, Dembek C (2011). Effects of cohort selection on the results of cost-effectiveness analysis of disease-modifying drugs for relapsing-remitting multiple sclerosis. *Journal of Managed Care Pharmacy*.

[35] Bell C, Graham J, Earnshaw S, Oleen-Burkey M, Castelli-Haley J, Johnson K (2007). Cost-effectiveness of four immunomodulatory therapies for relapsing-remitting multiple sclerosis: a Markov model based on long-term clinical data. *Journal of Managed Care Pharmacy*.

[36] Boggild M, Palace J, Barton P (2009). Multiple sclerosis risk sharing scheme: two year results of clinical cohort study with historical comparator. *British Medical Journal*.

[37] Brown MG, Jock Murray T, Sketris IS (2000). Cost-effectiveness of interferon beta-1b in slowing multiple sclerosis disability progression: first estimates. *International Journal of Technology Assessment in Health Care*.

[38] Castelli-Haley J, Oleen-Burkey MA, Lage MJ, Johnson KP (2009). Glatiramer acetate and interferon beta-1b: a study of outcomes among patients with multiple sclerosis. *Advances in Therapy*.

[39] Chiao E, Meyer K (2009). Cost effectiveness and budget impact of natalizumab in patients with relapsing multiple sclerosis. *Current Medical Research and Opinion*.

[40] Chilcott J, McCabe C, Tappenden P (2003). Modelling the cost effectiveness of interferon beta and glatiramer acetate in the management of multiple sclerosis. *British Medical Journal*.

[41] Clegg A, Bryant J (2001). Immunomodulatory drugs for multiple sclerosis: a systematic review of clinical and cost effectiveness. *Expert Opinion on Pharmacotherapy*.

[42] Clegg A, Bryant J, Milne R (2000). Disease-modifying drugs for multiple sclerosis: a rapid and systematic review. *Health Technology Assessment*.

[43] Coles A (2007). The fragile benefit of BENEFIT. *Lancet Neurology*.

[44] Compston A (2010). Commentary: scheme has benefited patients. *British Medical Journal*.

[45] Curtiss FR (2007). Pharmacoeconomic modeling of drug therapies for multiple sclerosis—are we building houses on sand?. *Journal of Managed Care Pharmacy*.

[46] Detournay B (2002). The value of economic modeling studies in the evaluation of treatment strategies for multiple sclerosis. *Value in Health*.

[47] Devlin N, Appleby J, Parkin D (2003). Patients' views of explicit rationing: what are the implications for health service decision-making?. *Journal of Health Services Research and Policy*.

[48] Earnshaw SR, Graham J, Oleen-Burkey M, Castelli-Haley J, Johnson K (2009). Cost effectiveness of glatiramer acetate and natalizumab in relapsing-remitting multiple sclerosis. *Applied Health Economics and Health Policy*.

[49] Ebers GC (2010). Commentary: outcome measures were flawed. *British Medical Journal*.

[50] Ellis SJ (2002). Bad decision NICE. *The Lancet*.

[51] Forbes RB, Lees A, Waugh N, Swingler RJ (1999). Population based cost utility study of interferon beta-1b in secondary progressive multiple sclerosis. *British Medical Journal*.

[52] Gani R, Giovannoni G, Bates D, Kemball B, Hughes S, Kerrigan J (2008). Cost-effectiveness analyses of natalizumab (Tysabri®) compared with other disease-modifying therapies for people with highly active relapsing-remitting multiple sclerosis in the UK. *PharmacoEconomics*.

[53] Gonsette RE (2004). A comparison of the benefits of mitoxantrone and other recent therapeutic approaches in multiple sclerosis. *Expert Opinion on Pharmacotherapy*.

[54] Greenhalgh J, Ford H, Long AF, Hurst K (2004). The MS Symptom and Impact Diary (MSSID): psychometric evaluation of a new instrument to measure the day to day impact of multiple sclerosis. *Journal of Neurology, Neurosurgery and Psychiatry*.

[55] Guo S, Bozkaya D, Ward A (2009). Treating relapsing multiple sclerosis with subcutaneous versus intramuscular interferon-beta-1a: modelling the clinical and economic implications. *PharmacoEconomics*.

[56] Heller JG (2002). Will public health survive QALYs?. *Canadian Journal of Clinical Pharmacology*.

[57] Herndon RM, Jacobs L (1998). Interferons should be used to treat most patients with MS. *Archives of Neurology*.

[58] Iskedjian M, Walker JH, Gray T, Vicente C, Einarson TR, Gehshan A (2005). Economic evaluation of Avonex (interferon beta-la) in patients following a single demyelinating event. *Multiple Sclerosis*.

[59] Jankovic SM, Kostic M, Radosavljevic M (2009). Cost-effectiveness of four immunomodulatory therapies for relapsing-remitting multiple sclerosis: a Markov model based on data a Balkan country in socioeconomic transition. *Vojnosanitetski Pregled*.

[60] Johnson KP, Due DL (2009). Benefits of glatiramer acetate in the treatment of relapsing-remitting multiple sclerosis. *Expert Review of Pharmacoeconomics and Outcomes Research*.

[61] Kendrick M, Johnson KI (2000). Long term treatment of multiple sclerosis with interferon-*β* may be cost effective. *PharmacoEconomics*.

[62] Kobelt G, Berg J, Lindgren P, Jonsson B, Stawiarz L, Hillert J (2008). Modeling the cost-effectiveness of a new treatment for MS (natalizumab) compared with current standard practice in Sweden. *Multiple Sclerosis*.

[63] Kobelt G, Jönsson L, Henriksson F, Fredrikson S, Jönsson B (2000). Cost-utility analysis of interferon beta-1b in secondary progressive multiple sclerosis. *International Journal of Technology Assessment in Health Care*.

[64] Kobelt G, Jönsson L, Miltenburger C, Jönsson B (2002). Cost-utility analysis of interferon beta-1b in secondary progressive multiple sclerosis using natural history disease data. *International Journal of Technology Assessment in Health Care*.

[65] Lapé Nixon M, Matud J, Yeh C, Prince HE (2009). Evaluation of a multiplex bead-based screening assay for detection of binding antibodies to interferon-beta. *Journal of Neuroimmunology*.

[66] Lazzaro C, Bianchi C, Peracino L, Zacchetti P, Uccelli A (2009). Economic evaluation of treating clinically isolated syndrome and subsequent multiple sclerosis with interferon *β*-1b. *Neurological Sciences*.

[67] Lie RK (2004). Research ethics and evidence based medicine. *Journal of Medical Ethics*.

[68] MacDonald R (2000). Cost effectiveness of multiple sclerosis drugs remains unknown. *British Medical Journal*.

[69] McCabe C, Chilcott J, Claxton K (2010). Continuing the multiple sclerosis risk sharing scheme is unjustified. *British Medical Journal*.

[70] McKee L (1998). Interferon beta produces only small benefits in multiple sclerosis. *British Medical Journal*.

[71] McNamee P (2007). What difference does it make? The calculation of QALY gains from health profiles using patient and general population values. *Health Policy*.

[72] McNaughton H, Kayes N, McPherson K (2006). Interferon beta, PHARMAC, and political directives: in the best interests of people with multiple sclerosis?. *New Zealand Medical Journal*.

[73] Medina-Redondo F, Herrera-Carranza J, Sanabria C (2004). The efficiency and cost-utility ratio of interferon beta in the treatment of multiple sclerosis in Andalusia. *Revista de Neurologia*.

[74] Meyer CM, Phipps R, Cooper D, Wright A (2003). Pharmacy benefit forecast for a new interferon Beta-1a for the treatment of multiple sclerosis: development of a first-line decision tool for pharmacy-budget planning using administrative claims data. *Journal of Managed Care Pharmacy*.

[75] Murdoch D, Lyseng-Williamson KA (2005). Subcutaneous recombinant interferon-*β*-1a (Rebif): a review of its use in relapsing-remitting multiple sclerosis. *Drugs*.

[76] Napier JC, Francis R, Wright G (2003). Shared scheme for assessing drugs for multiple sclerosis: cost effective provision of effective treatments for multiple sclerosis. *British Medical Journal*.

[77] Noyes K, Bajorska A, Chappel A (2011). Cost-effectiveness of disease-modifying therapy for multiple sclerosis: a population-based study. *Neurology*.

[78] Nuijten M, Mittendorf T (2010). A health-economic evaluation of disease-modifying drugs for the treatment of relapsing-remitting multiple sclerosis from the German societal perspective. *Clinical Therapeutics*.

[79] Nuijten MJC, Hutton J (2002). Cost-effectiveness analysis of interferon beta in multiple sclerosis: a markov process analysis. *Value in Health*.

[80] O' Day K, Meyer K, Miller RM, Agarwal S, Franklin M (2011). Cost-effectiveness of natalizumab versus fingolimod for the treatment of relapsing multiple sclerosis. *Journal of Medical Economics*.

[81] Parkin D, Jacoby A, McNamee P, Miller P, Thomas S, Bates D (2000). Treatment of multiple sclerosis with interferon *β*: an appraisal of cost-effectiveness and quality of life. *Journal of Neurology Neurosurgery and Psychiatry*.

[82] Parkin D, McNamee P, Jacoby A, Miller P, Thomas S, Bates D (1998). A cost-utility analysis of interferon beta for multiple sclerosis. *Health Technology Assessment*.

[83] Pickin M, Cooper CL, Chater T (2009). The multiple sclerosis risk sharing scheme monitoring study—early results and lessons for the future. *BMC Neurology*.

[84] Prosser LA, Kuntz KM, Bar-Or A, Weinstein MC (2004). Cost-effectiveness of interferon beta-1a, interferon beta-1b, and glatiramer acetate in newly diagnosed non-primary progressive multiple sclerosis. *Value in Health*.

[85] Pryse-Phillips W (2002). Newer long-term treatments for multiple sclerosis. *Clinical Neurology and Neurosurgery*.

[86] Raftery J (2010). Multiple sclerosis risk sharing scheme: a costly failure. *BMBritish Medical Journal*.

[87] Rawson N (2006). Response to McNaughton and colleagues regarding their article–interferon beta, PHARMAC, and political directives: in the best interests of people with multiple sclerosis?. *The New Zealand Medical Journal*.

[88] Reynolds MW, Stephen R, Seaman C, Rajagopalan K (2010). Healthcare resource utilization following switch or discontinuation in multiple sclerosis patients on disease modifying drugs. *Journal of Medical Economics*.

[89] Rich SJ, Meyer C (2003). Shortcomings in pharmacy benefit forecasting–interferon beta products. *Journal of Managed Care Pharmacy*.

[90] Richards RG (1996). Interferon beta in multiple sclerosis. *British Medical Journal*.

[91] Richards RG (2010). MS risk sharing scheme. Some clarification needed. *British Medical Journal*.

[92] Rieckmann P (2001). Early multiple sclerosis therapy in the effects of public health economics. *Medizinische Klinik*.

[93] Rieckmann P, Toyka KV (2002). Immunomodulatory staged therapy of multiple sclerosis. New aspects and practical applications, March 2002. *Nervenarzt*.

[94] Rubio-Terrés C, Arístegui Ruiz I, Medina Redondo F, Izquierdo Ayuso G (2003). Cost-utility analysis of multiple sclerosis treatment with glatiramer acetate or interferon beta in Spain. *Farmacia Hospitalaria*.

[95] Rubio-Terres C, Dominguez-Gil Hurle A (2005). Cost-utility analysis of relapsing-remitting multiple sclerosis treatment with azathioprine or interferon beta in Spain. *Revista de Neurología*.

[96] Sanchez-De la Rosa R, Sabater E, Casado MA (2011). Budget impact analysis of the first-line treatment of relapsing remitting multiple sclerosis in Spain. *Revista de Neurología*.

[97] Scolding N (2010). The multiple sclerosis risk sharing scheme. *British Medical Journal*.

[98] Stock G, Horowski R (2001). A short history of beta-interferon therapy of multiple sclerosis. *Medizinische Klinik*.

[99] Tappenden P, Chilcott JB, Eggington S, Oakley J, McCabe C (2004). Methods for expected value of information analysis in complex health economic models: developments on the health economics of interferon-*β* and glatiramer acetate for multiple sclerosis. *Health Technology Assessment*.

[100] Tappenden P, McCabe C, Chilcott J (2009). Cost-effectiveness of disease-modifying therapies in the management of multiple sclerosis for the medicare population. *Value in Health*.

[101] Tappenden P, Saccardi R, Confavreux C (2010). Autologous haematopoietic stem cell transplantation for secondary progressive multiple sclerosis: an exploratory cost-effectiveness analysis. *Bone Marrow Transplantation*.

[102] Thompson AJ (1999). Inequality in the availability of expensive treatments. *Journal of the Royal College of Physicians of London*.

[103] Tolley KH, Whynes DK (1997). Interferon-*β* in multiple sclerosis. Can we control its costs?. *PharmacoEconomics*.

[104] Touchette DR, Durgin TL, Wanke LA, Goodkin DE (2003). A cost-utility analysis of mitoxantrone hydrochloride and interferon beta-1b in the treatment of patients with secondary progressive or progressive relapsing multiple sclerosis. *Clinical Therapeutics*.

[105] Walley T (2004). Neuropsychotherapeutics in the UK: what Has Been the Impact of NICE on Prescribing?. *CNS Drugs*.

[106] Pan F, Goh JW, Cutter G, Su W, Pleimes D, Wang C (2012). Long-term cost-effectiveness model of interferon Beta-1b in the early treatment of multiple sclerosis in the United States. *Clinical Therapeutics*.

[107] Caloyeras JP, Zhang B, Wang C (2012). Cost-effectiveness analysis of interferon beta-1b for the treatment of patients with a first clinical event suggestive of multiple sclerosis. *Clinical Therapeutics*.

[108] Kobelt G, Texier-Richard B, Lindgren P (2009). The long-term cost of multiple sclerosis in France and potential changes with disease-modifying interventions. *Multiple Sclerosis*.

[109] Perini P, Calabrese M, Tiberio M, Ranzato F, Battistin L, Gallo P (2006). Mitoxantrone versus cyclophosphamide in secondary-progressive multiple sclerosis: a comparative study. *Journal of Neurology*.

[110] Goodin DS, Frohman EM, Garmany GP (2002). Disease modifying therapies in multiple sclerosis: report of the therapeutics and technology assessment subcommittee of the American academy of neurology and the MS council for clinical practice guidelines. *Neurology*.

[111] National Collaborating Centre for Chronic Conditions (2003). Multiple sclerosis: management of multiple sclerosis in primary and secondary care.

[112] Weinstein MC, O’Brien B, Hornberger J (2003). Principles of good practice for decision analytic modeling in health-care evaluation: report of the ISPOR task force on good research practices—modeling studies. *Value in Health*.

